# Wavelet Autoregulation Monitoring Identifies Blood Pressures Associated With Brain Injury in Neonatal Hypoxic-Ischemic Encephalopathy

**DOI:** 10.3389/fneur.2021.662839

**Published:** 2021-04-28

**Authors:** Xiuyun Liu, Aylin Tekes, Jamie Perin, May W. Chen, Bruno P. Soares, An N. Massaro, Rathinaswamy B. Govindan, Charlamaine Parkinson, Raul Chavez-Valdez, Frances J. Northington, Ken M. Brady, Jennifer K. Lee

**Affiliations:** ^1^Department of Anesthesiology and Critical Care Medicine, Johns Hopkins University, Baltimore, MD, United States; ^2^Department of Radiology, Johns Hopkins University, Baltimore, MD, United States; ^3^Department of Pediatrics, Center for Child and Community Health Research, Johns Hopkins University, Baltimore, MD, United States; ^4^Division of Neonatology, Johns Hopkins University, Baltimore, MD, United States; ^5^Department of Radiology, University of Vermont, Burlington, VT, United States; ^6^Fetal Medicine Institute, Children's National Health System, Washington, DC, United States; ^7^The George Washington University School of Medicine, Washington, DC, United States; ^8^Division of Neonatology, Children's National Health System, Washington, DC, United States; ^9^Department of Anesthesiology, Lurie Children's Hospital, Chicago, IL, United States

**Keywords:** newborn, hypoxia, cerebrovascular circulation, brain, hypothermia, cerebral autoregulation

## Abstract

Dysfunctional cerebrovascular autoregulation may contribute to neurologic injury in neonatal hypoxic-ischemic encephalopathy (HIE). Identifying the optimal mean arterial blood pressure (MAPopt) that best supports autoregulation could help identify hemodynamic goals that support neurologic recovery. In neonates who received therapeutic hypothermia for HIE, we hypothesized that the wavelet hemoglobin volume index (wHVx) would identify MAPopt and that blood pressures closer to MAPopt would be associated with less brain injury on MRI. We also tested a correlation-derived hemoglobin volume index (HVx) and single- and multi-window data processing methodology. Autoregulation was monitored in consecutive 3-h periods using near infrared spectroscopy in an observational study. The neonates had a mean MAP of 54 mmHg (standard deviation: 9) during hypothermia. Greater blood pressure above the MAPopt from single-window wHVx was associated with less injury in the paracentral gyri (*p* = 0.044; *n* = 63), basal ganglia (*p* = 0.015), thalamus (*p* = 0.013), and brainstem (*p* = 0.041) after adjustments for sex, vasopressor use, seizures, arterial carbon dioxide level, and a perinatal insult score. Blood pressure exceeding MAPopt from the multi-window, correlation HVx was associated with less injury in the brainstem (*p* = 0.021) but not in other brain regions. We conclude that applying wavelet methodology to short autoregulation monitoring periods may improve the identification of MAPopt values that are associated with brain injury. Having blood pressure above MAPopt with an upper MAP of ~50–60 mmHg may reduce the risk of brain injury during therapeutic hypothermia. Though a cause-and-effect relationship cannot be inferred, the data support the need for randomized studies of autoregulation and brain injury in neonates with HIE.

## Introduction

Nearly one million neonatal deaths worldwide each year are related to hypoxic-ischemic encephalopathy (HIE) from intrapartum complications (1). Though therapeutic hypothermia has reduced mortality, additional treatments are needed to prevent the persistent moderate-to-severe neurologic disabilities that affect almost half of survivors who receive this therapy (2). Alterations in cerebrovascular blood pressure autoregulation, which holds blood flow stable across changes in perfusion pressure, may contribute to HIE-related brain injury. Neuroprotective hemodynamic goals for infants with HIE are unclear, and many clinicians base their blood pressure goals on the neonate's gestational age in weeks +5 (3). However, maintaining mean arterial blood pressure (MAP) above the gestational age+5 does not reduce neurologic injury in infants with HIE (4).

Autoregulation monitoring has the potential to clarify hemodynamic goals during recovery from brain injury. Blood pressures below the optimal MAP (MAPopt) at which autoregulation is most robust are associated with greater brain injury on MRI and worse neurocognitive outcomes in neonates with HIE (4–7). The pediatric traumatic brain injury guidelines recommend that clinicians consider monitoring autoregulation to reduce secondary injury (8). Unfortunately, similar recommendations cannot be made in neonatal medicine where autoregulation monitoring is challenged by the reliance on non-invasive cranial methods that often have high signal variability. More reliable techniques with less signal noise must be developed for neonatal autoregulation monitoring.

Mathematical algorithms that determine the relationship between blood pressure and cerebral blood volume can be used to assess autoregulation. The relative total tissue hemoglobin (rTHb) from near-infrared spectroscopy (NIRS) is a surrogate measure of cerebral blood volume during autoregulatory vasoreactivity (9). In theory, changes in cerebral metabolic rate, such as from temperature modulation, should have little effect on rTHb because rTHb measures oxygenated and deoxygenated hemoglobin. Indices derived from rTHb accurately measure autoregulation during therapeutic hypothermia (10).

The correlation between MAP and rTHb generates the hemoglobin volume index (HVx) (10). Though correlation is commonly used to assess autoregulation (4, 11), it can have considerable signal variability when applied to short monitoring periods. We therefore developed the wavelet hemoglobin volume index (wHVx) (12, 13) and a multi-window data processing method (14) to produce a more stable autoregulation index suitable for monitoring short time windows. Whether wHVx and multi-window processing can identify blood pressures associated with HIE brain injury is unknown. In this study, we tested wHVx and correlation HVx with single- and multi-window data processing in newborns with HIE. We hypothesized that blood pressure deviation from the MAPopt identified by wHVx would be associated with regional and global brain injury on MRI. We secondarily tested the MAPopt from correlation HVx and whether the multi-window technique improves MAPopt identification relative to the single-window technique.

## Materials and Methods

We measured autoregulatory vasoreactivity using NIRS in neonates who received therapeutic hypothermia for HIE in the Johns Hopkins neonatal intensive care unit (NICU). This prospective, observational study was approved by the Johns Hopkins Institutional Review Board. Before May 2013, written consent was obtained from neonates' parents for study participation. After that, NIRS became standard clinical care for HIE in our NICU, and the IRB waived the requirement for written consent. Sixty-six neonates in this study were reported in at least one of our past reports (4–7, 15–18). Four neonates contributed new data to the study.

### Enrollment Criteria and Clinical Care

All neonates diagnosed with HIE were screened between September 2010 and November 2015. Study enrollment criteria included the receipt of therapeutic hypothermia, continuous arterial blood pressure monitoring, gestational age ≥35 weeks, and birth weight ≥1,800 g. Neonates without an arterial blood pressure cannula, who did not receive hypothermia, or who were transferred to another intensive care unit for potential extracorporeal membrane oxygenation were not eligible for the study.

Neonates were diagnosed with moderate or severe HIE at their birth hospital based on the National Institute of Child Health and Human Development (NICHD) Neonatal Research Network criteria as previously described (7, 19). Outborn neonates started passive cooling and were transported to our NICU for active therapeutic hypothermia. A modified Sarnat encephalopathy score (19, 20) was determined when they arrived at our NICU. Five neonates who were initially diagnosed with moderate HIE at the outside hospital were subsequently graded to have mild HIE at our institution during active cooling. The clinicians continued therapeutic hypothermia because perinatal acidosis increases the risk of persistent brain injury, even in mild encephalopathy (21), and the encephalopathy may rapidly evolve (22). These neonates met study criteria and therefore were enrolled.

We have reported our therapeutic hypothermia protocol (4, 6). Briefly, neonates underwent whole-body cooling to a goal rectal temperature of 33.5 ± 0.5°C for 72 h followed by rewarming at ≤ 0.5°C/h. For the study, the rewarming period was defined by a temperature increase from 34.1 to 36.4°C. The normothermia period was defined by a temperature ≥36.5°C. Clinicians followed routine clinical practice for blood pressure management, and they had access to the NIRS regional cerebral oxyhemoglobin data but not the HVx or wHVx. All neonates received morphine for sedation followed by as-needed fentanyl, hydromorphone, clonidine, or benzodiazepines. Dopamine was administered for hypotension, followed by dobutamine or epinephrine at the clinicians' discretion. Seizures were diagnosed by electroencephalogram and treated with phenobarbital. If the seizures persisted, neonates were administered levetiracetam, fosphenytoin, or topiramate. Hydrocortisone was administered for adrenal suppression or hypotension that was refractory to vasopressors. The neonates' partial pressure of arterial carbon dioxide (PaCO_2_) levels were categorized into one of four categories: (1) all 35–45 mmHg; (2) some <35 mmHg but none >45 mmHg; (3) none <35 mmHg but some >45 mmHg; and (4) some <35 mmHg and some >45 mmHg (4, 23).

We assigned each neonate a perinatal insult score based on common clinical criteria, including whether delivery was emergent, Apgar score at 10 min, first blood gas pH and base deficit, Sarnat stage, and need for mechanical ventilation (4, 24) (Supplementary Table 1). For outborn neonates, we used the Sarnat encephalopathy score that was obtained upon their arrival at our NICU. The purpose of the perinatal insult score, which is independently associated with HIE brain injury (24), is to provide a single variable that describes the neonate's clinical status soon after birth for multivariate analysis (4, 24). The investigator who assigned the perinatal insult scores (RC-V) was blinded to the autoregulation and MRI data.

### Autoregulation Monitoring

**Figure 1:**
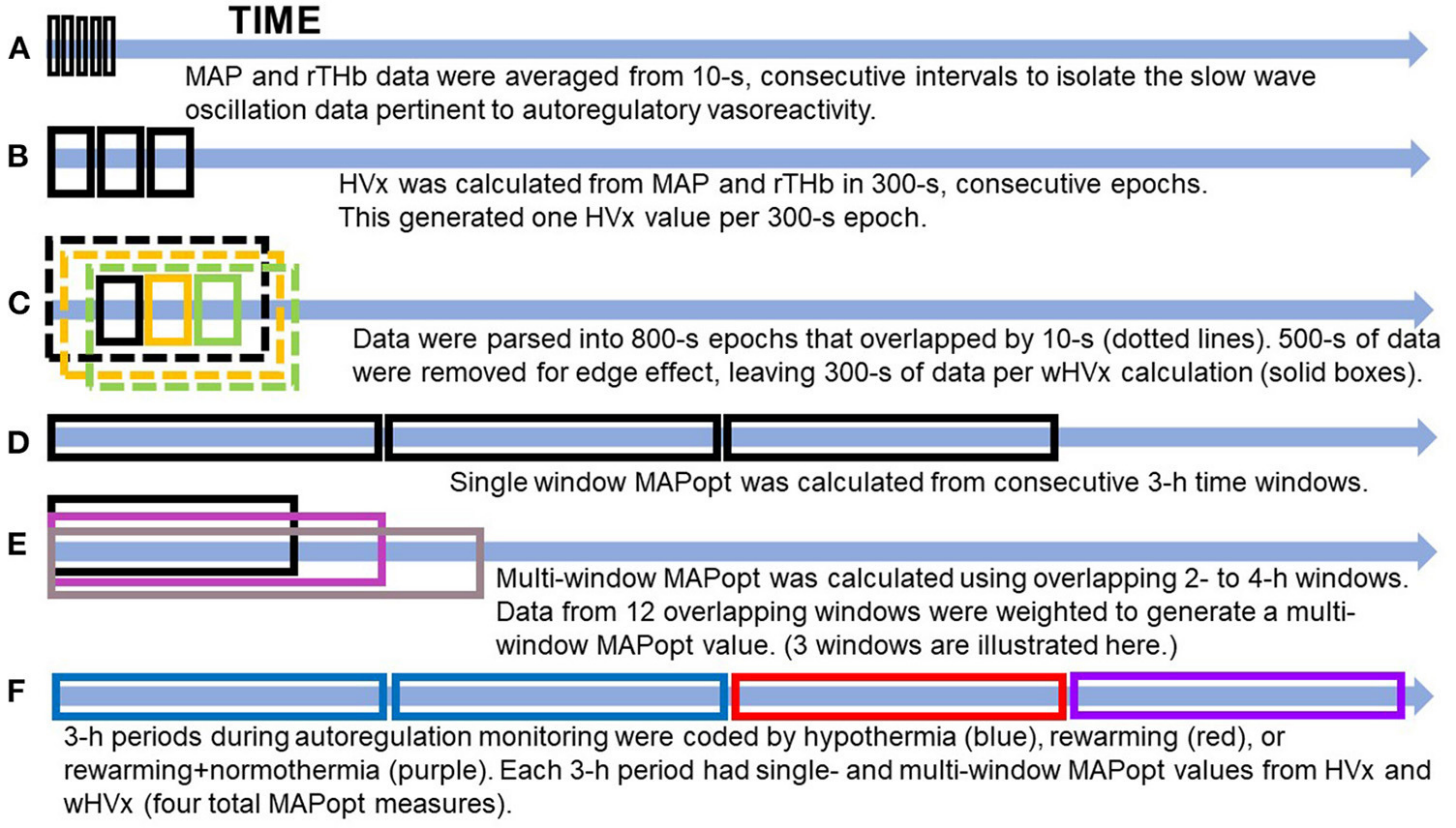
Illustration of data processing. **(A)** The data were filtered to remove high-frequency waveforms, including those associated with pulse and respiration. **(B)** The hemoglobin volume index (HVx). **(C)** The wavelet hemoglobin volume index (wHVx). **(D)** Single-window, optimal mean arterial blood pressure (MAPopt). **(E)** Multi-window MAPopt. **(F)** MAPopt values were calculated in consecutive 3-hour periods that were coded by the neonate's core temperature. Boxes are not to scale. MAP, mean arterial blood pressure; rTHb, NIRS relative total tissue hemoglobin.

A bedside laptop computer continuously monitored MAP from the arterial cannula and rTHb from bilateral cerebral NIRS (INVOS 5100; neonatal probes; Medtronic, Minneapolis, MN) during hypothermia, rewarming, and the first 3 h of normothermia. Data were sampled at 100 Hz with ICM+ software (University of Cambridge, Cambridge Enterprise, Cambridge, UK, https://icmplus.neurosurg.cam.ac.uk). The rTHb was detected by NIRS to obtain a surrogate measure of cerebral blood volume (9). (Additional information is provided in the Supplementary Material.) Artifacts in the arterial blood pressure tracing, including those from flushes and transducer adjustments, were filtered out manually. We averaged the MAP and rTHb data in consecutive, 10-s intervals to exclude high-frequency waveforms from respiration and pulse (Figure 1A). This filtering technique leaves slow-wave oscillation data from autoregulatory vasoreactivity (9). An investigator (XL) blinded to the neonate's temperature and brain MRI measures processed all of the autoregulation data.

### HVx Calculation

HVx was calculated as the Pearson correlation coefficient between 10-s averages of MAP and rTHb from 30 paired samples in consecutive, 300-s epochs (Figure 1B). HVx is a continuous metric of autoregulatory vasoreactivity that ranges from −1 to +1. Negative or near-zero HVx indicates functional autoregulation because MAP and cerebral blood volume correlate negatively or not at all. When autoregulation becomes dysfunctional, HVx becomes increasingly positive and approaches +1 because MAP and cerebral blood volume correlate (10).

### wHVx Calculation

Using methodology that we previously validated in piglets (12, 13), we calculated the wavelet transform phase shift between MAP and rTHb in the frequency range of 0.007–0.05 Hz. Intracranial slow waves from autoregulatory vasoreactivity are known to occur in this frequency range (9, 25). In brief, we calculated the wavelet transform phase shift at each scale-frequency point in 800-s data segments using a moving time window that was updated every 10 s and a wavelet transform coherence threshold of 0.46 (26) (Supplementary Material). This method assumes that data with good coherence represent true physiology whereas poorly coherent data are from signal noise.

Then, we calculated the wavelet semblance (the cosine of wavelet phase shift) to generate the wHVx. This process produced several wavelet semblances from 0.007 to 0.05 Hz at each time point. The wavelet semblance values were then averaged along the frequency domain to create one wHVx value for each time point. We chose an 800-s window length because an edge effect necessitated that we reject approximately 500 s of data (27), leaving 300 s of data to calculate wHVx. Therefore, both the wHVx and correlation HVx data were analyzed in 300-s epochs (Figure 1C) (Supplementary Material).

The wHVx is a continuous autoregulatory vasoreactivity index that ranges from −1 to +1. Functional autoregulation generates a high phase shift owing to the inverse correlation between MAP and rTHb (28). For example, when autoregulation is functional, increases in blood pressure cause the cerebral arterioles to constrict with a reduction in cerebral blood volume that is detected by rTHb. Perfect autoregulation causes a 180° phase shift between MAP and rTHb, thereby generating a wHVx of −1. When autoregulation is dysfunctional, increases in blood pressure cause the cerebral blood volume and rTHb to rise also. Complete pressure passivity generates a 0° phase shift between MAP and rTHb with a wHVx of +1. Thus, the wHVx and correlation HVx indices have the same directionality. Functional autoregulation is indicated by negative or near-zero wHVx and HVx. Dysfunctional autoregulation causes wHVx and HVx to become positive.

### Identification of MAPopt

**Figure 2:**
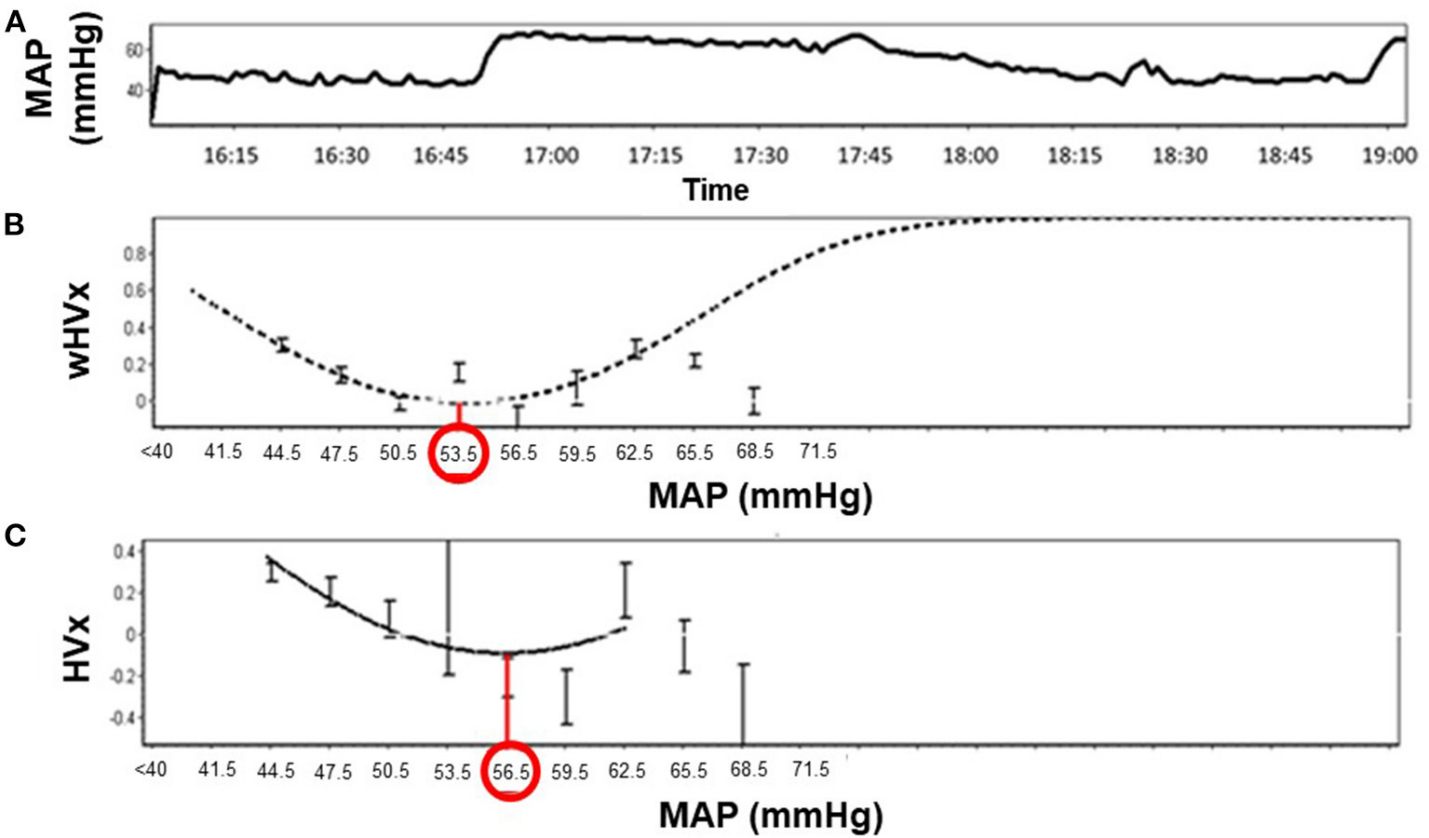
Identification of optimal mean arterial blood pressure (MAPopt) by the hemoglobin volume index (HVx) and the wavelet hemoglobin volume index (wHVx) in an example neonate. **(A)** Mean arterial blood pressure; **(B)** Using wHVx, the curve fitting method identified MAPopt at 53.5 mmHg. **(C)** HVx identified MAPopt at 56.5 mmHg. The curved lines show the optimal curve fit applied by ICM+ software. The bars are standard deviations.

Graphs were generated by sorting each neonate's MAP into 3-mmHg bins on the x-axis and the mean HVx or wHVx of each bin on the y-axis. An automatic curve fitting method that used the smallest curve fitting error in ICM+ (13) generated a U-shaped curve with MAPopt at the nadir (Figure 2). Neonates without a nadir were coded as having an unidentifiable MAPopt.

We derived MAPopt from HVx and wHVx using single- and multi-window methods to generate four comparison indices. Single-window MAPopt was calculated in consecutive 3-h windows to generate one MAPopt per window (Figures 1D, 2). Thus, every neonate had many single-window MAPopt values from HVx and wHVx across time.

The multi-window MAPopt was calculated by using a previously published method (14). Twelve 2-h to 4-h overlapping windows of data were updated every 10 min (Figure 1E) to generate 12 MAP-HVx and 12 MAP-wHVx graphs. A weighting process was applied based on curve fit error, curve shape, and window duration. Data with smaller curve fit errors that formed U-shapes and that were from shorter time windows were weighted heavily and contributed more to the MAPopt calculation (14). The weighted average from the 12 graphs generated the final multi-window MAPopt values from HVx and wHVx across time.

Finally, each neonate's autoregulation recording was divided into consecutive 3-h periods from the beginning of the recording. These were coded according to the neonate's rectal temperature (Figure 1F). Temperatures ≤ 34.0°C were considered hypothermia, and the rewarming period consisted of temperatures 34.1°C to 36.4°C. The rewarming+normothermia period included at least 15 min of rewarming plus normothermia, which was defined as ≥36.5°C. We did not analyze periods that contained a mixture of hypothermia and rewarming or only normothermia. One investigator (JKL) who was blinded to the autoregulation and MRI data coded the temperatures for each period. Ultimately, each neonate had four MAPopt values calculated by the single- and multi-window methods from HVx and wHVx in each 3-h period across hypothermia, rewarming, and the transition between rewarming and normothermia.

### Brain MRI

Brain MRIs were obtained in neonates between 4 and 16 days of life on 1.5T (Avanto; Siemens, Erlangen, Germany) or 3T (Avanto; Siemens) clinical MRI scanners. All neonates were imaged without anesthesia during natural sleep. We reported our MRI methods previously (5–7). The neuroradiologists were blinded to the autoregulation indices and blood pressure data. An experienced pediatric neuroradiologist (AT) scored each MRI qualitatively as no, mild, moderate, or severe injury using T1, T2, and diffusion tensor imaging (DTI) in six regions: paracentral gyri, global white matter, thalamus, basal ganglia, posterior limb of the internal capsule, and brainstem (4).

Two pediatric neuroradiologists (AT, BS) also graded the T1 and T2 MRIs using the NICHD Neonatal Research Network score (2). This is a standardized score with categories of 0: no injury; **1A**: minimal cerebral lesions and no injury in the thalamus, basal ganglia, or internal capsule; **1B**: more extensive cerebral lesions without injury in the thalamus, basal ganglia, or internal capsule and no infarction; **2A**: any injury in thalamus, basal ganglia, or anterior or posterior limb of the internal capsule or watershed infarction; **2B**: same criteria as category **2A** with additional cerebral lesions; and **3**: cerebral hemispheric devastation. To test agreement in grading the MRIs, the two neuroradiologists independently interpreted 10 MRIs.

### Statistical Analysis

Statistical analysis was conducted with R (www.r-project.org/). We used proportional odds logistic regression to examine the associations between autoregulation and brain injury in data stratified by three temperatures: hypothermia, rewarming, and rewarming + normothermia. Autoregulation was measured in each neonate by the maximal blood pressure above or below MAPopt. We also measured the area under the curve (AUC; min × mmHg/h) of time (minutes) with blood pressure above or below MAPopt and blood pressure (mmHg) above or below MAPopt normalized to the monitoring duration (hours) (4). These autoregulation measures were obtained in each 3-h period coded by temperature.

The associations between each neonate's autoregulation and brain injury parameters were additionally analyzed with adjustments for sex, PaCO_2_ level, perinatal insult score, vasopressor use, and presence of electroencephalographic seizures. We selected these covariates because of their potential associations with autoregulation and brain injury (15, 23, 29–33).

We adjusted for multiple comparisons using Bonferroni corrections within each temperature and brain injury metric. For example, when analyzing the maximal blood pressure above MAPopt during hypothermia, 24 tests compared MAPopt from single- and multi-window wHVx and correlation HVx (four types of MAPopt) in 6 brain regions. Four additional tests compared maximal blood pressure above MAPopt with the NICHD global brain injury score. For the entire study encompassing the three temperatures (hypothermia, rewarming, and rewarming + normothermia), we performed 336 unadjusted and 336 adjusted comparisons. The Bonferroni correction controlled for the inflation of type 1 error caused by testing different MAPopt measures across time and in multiple brain regions (34). The 95% confidence intervals of the regression coefficients and the *p-*values were adjusted for multiple comparisons. *p* < 0.05 was considered statistically significant.

### Sample Size Estimation

This is a secondary and exploratory analysis of data from our prior studies (4–7, 15–18). No other studies have calculated MAPopt using wavelet or multi-window autoregulation monitoring in HIE to our knowledge. Nonetheless, a separate study of HIE using a different wavelet autoregulation index showed that an approximate mean index difference of ≥0.25 identified neonates who died or suffered brain injury (35). Using these estimates, a sample of 17 newborns with brain injury and 17 without injury would have power >0.80 at the alpha 0.05 level (PS Power and Sample Size Calculations, v. 3.0) (36, 37).

## Results

**Figure 3:**
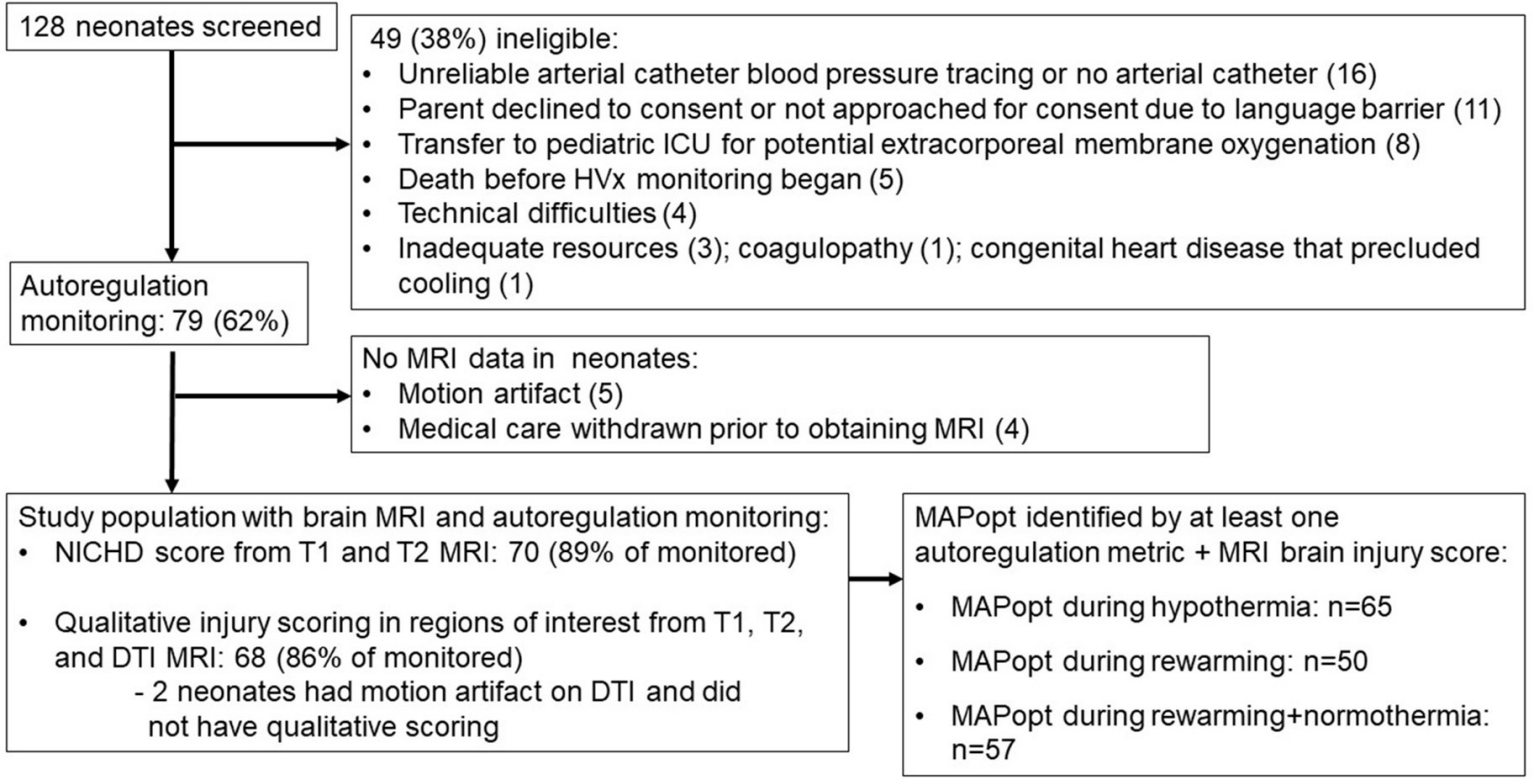
Study screening and enrollment. ICU, intensive care unit; MRI, magnetic resonance imaging; NICHD, National Institute of Child Health and Human Development; DTI, diffusion tensor imaging; MAPopt, optimal mean arterial blood pressure.

Seventy-nine neonates were enrolled for autoregulation monitoring, and 70 (89%) received a brain MRI (Figure 3). The majority were delivered emergently by unscheduled cesarean section for documented fetal distress (Table 1). Autoregulation was monitored for a mean total of 54.4 h [standard deviation (SD): 21.7 h; 95% confidence interval (CI): 49.5, 59.1 h] in all neonates.

**Table 1:** Clinical descriptions of neonates (*n* = 79).

**Characteristic**	**Mean (SD) or *n* (%)**
Male sex	47 (59%)
Gestational age, weeks	39.1 (1.5)
Emergency delivery^a^	56 (71%)
Cesarean section	60 (76%)
10-min Apgar score	4.8 (2.2)
Vasopressor use	52 (66%)
Seizures^b^	31 (39%)
Required mechanical ventilation	41 (52%)
Sarnat encephalopathy score^c^	
1	5 (6%)
2	59 (75%)
3	15 (19%)
PaCO_2_, mmHg	
All 35–45	6 (8%)
Some <35, all <45	16 (20%)
None <35, some >45	31 (39%)
Some <35, some >45	26 (33%)
pH of first arterial blood gas^d^	7.10 (0.16)
Base deficit of first arterial blood gas^e^	−16.1 (7.4)
Perinatal insult score	6 (1.4)

a *Defined as an unscheduled cesarean delivery for fetal distress*.

b *Diagnosed by electroencephalography*.

c *For neonates born at an outside hospital, the Sarnat score obtained after arrival to the Johns Hopkins neonatal intensive care unit is reported*.

d *Seventy seven neonates had pH values from their first arterial blood gas*.

e *Fifty four neonates had base deficit values from their first arterial blood gas*.

*PaCO_2_, partial pressure of arterial carbon dioxide*.

The neuroradiologists had 100% agreement in grading injury on 10 MRIs. Most neonates had mild or no injury in the paracentral gyri, white matter, basal ganglia, thalamus, posterior limb of the internal capsule, and brain stem, with NICHD scores of 0, **1A**, or **1B** (Table 2). The white matter most commonly showed injury, whether mild, moderate, or severe, whereas gray matter and brainstem more often had no injury. White matter was also the most common region to have severe injury.

**Table 2:** MRI interpretation by brain region and National Institute of Child Health and Human Development score.

**Brain region^a^**	***n* (%)**
**Paracentral gyri (*n* = 68)**	
No injury	41 (60)
Mild injury	14 (21)
Moderate injury	6 (9)
Severe injury	7 (10)
**White matter (*n* = 68)**	
No injury	15 (22)
Mild injury	29 (43)
Moderate injury	11 (16)
Severe injury	13 (19)
**Basal ganglia (*n* = 68)**	
No injury	32 (47)
Mild injury	19 (28)
Moderate injury	10 (15)
Severe injury	7 (10)
**Thalamus (*n* = 68)**	
No injury	30 (44)
Mild injury	18 (27)
Moderate injury	11 (16)
Severe injury	9 (13)
**Posterior limb of the internal capsule (*n* = 68)**	
No injury	46 (68)
Mild injury	11 (16)
Moderate injury	6 (9)
Severe injury	5 (7)
**Brainstem (*n* = 68)**	
No injury	32 (47)
Mild injury	19 (28)
Moderate injury	10 (15)
Severe injury	7 (10)
**NICHD score (*n* = 70)**	
0	37 (53)
1A	6 (9)
1B	12 (17)
2A	6 (9)
2B	8 (11)
3	1 (1)

a *Seventy neonates had National Institute of Child Health and Human Development (NICHD) brain injury scoring on T1 and T2 MRI. The regional injury score, which required interpretation of T1, T2, and diffusion tensor imaging (DTI) MRI, was completed in only 68 neonates because two had motion artifact on DTI*.

### Blood Pressure and MAPopt

Mean MAP was 54 mmHg (SD: 9; 95% CI: 52, 55) during hypothermia, 49 mmHg (SD: 8; 95% CI: 48, 50) during rewarming, and 49 mmHg (SD: 7; 95% CI: 48, 50) during rewarming+normothermia. Values for MAPopt differed by 0–3 mmHg between indices when all values were averaged across a temperature period (Table 3).

**Table 3:** Mean optimal arterial blood pressure values identified by the autoregulation metrics.

**Mathematical algorithm**	**Hypothermia mean (SD)**		**Rewarming mean (SD)**		**Rewarming + normothermia mean (SD)**	
	***n***	**mmHg**	***n***	**mmHg**	***n***	**mmHg**
MAPopt_HVx (SW)	75	52 (6)	48	51 (9)	45	51 (8)
MAPopt_wHVx (SW)	74	52 (6)	45	52 (9)	47	54 (8)
MAPopt_HVx (MW)	76	53 (6)	50	51 (8)	52	51 (8)
MAPopt_wHVx (MW)	77	52 (5)	55	52 (7)	55	53 (7)

*SD, standard deviation; HVx, hemoglobin volume index; wHVx, wavelet hemoglobin volume index; SW, single window; MW, multiple window; MAPopt, optimal mean arterial blood pressure*.

### Wavelet HVx

Sixty-three neonates with MAPopt derived from single-window wHVx during hypothermia had regional brain injury scored on MRI. Greater maximal blood pressure above MAPopt was associated with less injury in the paracentral gyri (β = −0.147; 95% CI: −0.284, −0.010; *p* = 0.044), basal ganglia (β = −0.142; 95% CI: −0.261, −0.023; *p* = 0.015), thalamus (β= −0.149; 95% CI: −0.272, −0.026; *p* = 0.013), and brainstem (β = −0.120; 95% CI: −0.232, −0.009; *p* = 0.041) after adjusting for sex, vasopressors, seizures, PaCO_2_, and the perinatal insult score. An example coefficient interpretation would be that for every 1 mmHg increase in maximal blood pressure above MAPopt, the odds ratio of increased paracentral gyri injury was 0.863 [exp(−0.147)] per increase in injury level (e.g., mild to moderate).

Blood pressure below the single-window wHVx MAPopt and the AUC during hypothermia were not related to regional brain injury (*p* > 0.05 for all comparisons, Table 4). Blood pressure relative to this MAPopt during rewarming and rewarming + normothermia was also not associated with regional injury.

**Table 4:** Comparisons of blood pressure relative to the MAPopt from wHVx during hypothermia.

	**Maximal MAP above MAPopt (*n* = 63)**		**Maximal MAP below MAPopt (*n* = 63)**		**AUC above MAPopt (*n* = 63)**		**AUC below MAPopt (*n* = 63)**	
	**β**	***P***	**β**	***P***	**β**	***P***	**β**	***P***
**Single-window wHVx**								
Paracentral gyri	−0.147	**0.044**	−0.019	1.000	−0.003	1.000	−0.002	1.000
White matter	−0.075	0.473	−0.004	1.000	−0.002	1.000	0.000	1.000
Basal ganglia	−0.142	**0.015**	0.003	1.000	−0.005	0.446	0.002	1.000
Thalamus	−0.149	**0.013**	−0.011	1.000	−0.005	0.439	0.001	1.000
Posterior limb of the internal capsule	−0.064	1.000	0.010	1.000	−0.004	1.000	0.000	1.000
Brainstem	−0.120	**0.041**	0.006	1.000	−0.004	1.000	0.002	1.000
	**Maximal MAP above MAPopt (*n* = 65)**		**Maximal MAP below MAPopt (*n* = 65)**		**AUC above MAPopt (*n* = 65)**		**AUC below MAPopt (*n* = 65)**	
	**β**	***P***	**β**	***P***	**β**	***P***	**β**	***P***
**Multi-window wHVx**								
Paracentral gyri	−0.069	1.000	0.006	1.000	−0.004	1.000	0.001	1.000
White matter	−0.037	1.000	0.004	1.000	−0.002	1.000	0.000	1.000
Basal ganglia	−0.085	0.216	−0.012	1.000	−0.004	1.000	0.002	1.000
Thalamus	−0.089	0.194	−0.021	1.000	−0.005	1.000	0.001	1.000
Posterior limb of the internal capsule	−0.040	1.000	0.019	1.000	−0.003	1.000	0.001	1.000
Brainstem	−0.089	0.139	−0.017	1.000	−0.003	1.000	0.001	1.000

*Comparisons were adjusted for sex, partial pressure of arterial carbon dioxide, perinatal insult score, vasopressor use, and presence of electroencephalographic seizures. The analyses were also adjusted for multiple comparisons using Bonferroni corrections. Bold values are statistically significant p-values (p-values < 0.05)*.

*MAP, mean arterial blood pressure; MAPopt, optimal mean arterial blood pressure; wHVx, wavelet hemoglobin volume index; AUC, area under the curve*.

NICHD global brain injury scores were graded in 65 neonates with MAPopt derived from single-window wHVx during hypothermia. Greater maximal blood pressure above this MAPopt was associated with less injury in the univariate analysis (β = −0.084; 95% CI: −0.160, −0.008; *p* = 0.033) but not in the multivariate analysis. The NICHD injury score was not related to blood pressure below this MAPopt or the AUC during hypothermia nor with any blood pressure metric during rewarming or rewarming + normothermia. The multi-window wHVx did not identify any relationships between blood pressure, MAPopt, and brain injury.

### Correlation HVx

MAPopt during hypothermia was identified by the multi-window, correlation HVx in 64 neonates with regional brain injury measures. Higher blood pressure above this MAPopt was associated with less brainstem injury in the multivariate analysis (β = −0.117; 95% CI: −0.217, −0.009; *p* = 0.021). Blood pressure below this MAPopt and the AUC during hypothermia had no association with injury in any region. Moreover, regional brain injury was unrelated to blood pressure and MAPopt during rewarming or rewarming+normothermia. The NICHD score also was not associated with this MAPopt. Finally, single-window HVx did not identify any relationships between blood pressure, MAPopt, and brain injury.

## Discussion

Maintaining blood pressure within the range that optimizes autoregulation could mitigate brain injury in HIE (4–7, 16). We tested whether wHVx and correlation HVx, which we validated in piglets with HIE (13), and single- and multi-window methodology (14) identify MAPopt in short monitoring periods. Having blood pressure above the single-window wHVx-derived MAPopt during hypothermia was associated with less injury in the paracentral gyri, basal ganglia, thalamus, and brainstem after adjustments for sex, vasopressor use, seizures, PaCO_2_, and perinatal insult score. Blood pressure that exceeded the MAPopt from multi-window, correlation HVx was associated with reduced injury in the brainstem only. We conclude that single-window wavelet methodology may increase accuracy in identifying MAPopt values that are associated with brain injury when using short monitoring periods. In neonates with an upper MAP of approximately 50–60 mmHg, having blood pressure above the MAPopt identified by single-window wHVx might be able to reduce the risk of neurologic injury.

### MAPopt From Wavelet and Correlation HVx

Autoregulation is a frequency-dependent phenomenon. We theorized that our wavelet methodology, which is a time-frequency analysis, would detect physiologically relevant fluctuations in blood pressure and cerebral blood volume. Correlation HVx might detect signals that are unrelated to the frequency of autoregulation because it characterizes all of the frequency components. Accordingly, wHVx identified MAPopt values that were significantly related to brain injury in multiple regions, including the paracentral gyri, basal ganglia, thalamus, and brainstem. In contrast, correlation HVx identified MAPopt that was only associated with brainstem injury. The time-frequency analysis might be responsible for the superior performance of wHVx relative to correlation HVx during short monitoring periods.

Neonates with greater blood pressure above the wHVx MAPopt had less brain injury than did neonates with lower blood pressure. This outcome agrees with our past findings that less paracentral gyri injury is associated with blood pressure exceeding MAPopt derived from correlation HVx (4). The risk of intraventricular hemorrhage in premature neonates is also reduced when blood pressure exceeds the MAPopt derived from a tissue oxygenation heart rate reactivity index (38). Moreover, we previously demonstrated that blood pressure below MAPopt relates to greater brain injury (4–6, 16). Though these findings suggest that higher blood pressure may be beneficial, extreme caution must be taken given the developing brain's fragile vascular anatomy and unique vulnerability (39). The neonates' MAP in our study had an upper 95% CI limit of 55 mmHg. We estimate that with an upper MAP limit of 50–60 mmHg, hypotension below MAPopt may be more deleterious than blood pressure above MAPopt.

MAPopt showed no association with white matter injury, even though white matter was the most commonly injured region. This finding contrasts with our past studies, which showed a relationship between white matter injury and autoregulation in long monitoring durations (4–6). Studies in preterm neonates show a link between periventricular white matter injury and hypotension (40). Less is known about cerebral blood flow–blood pressure regulation in subcortical and deep white matter in near-term or term newborns with HIE. It is possible that the gray matter is more sensitive to blood pressure deviation from MAPopt. Therapies beyond hemodynamic support may be needed to reduce white matter injury in HIE. Moreover, the 4–16-day age range in which the MRIs were obtained could have influenced the results because the neonates were imaged at different stages of evolving injury.

### Single- and Multi-Window

We also tested single- and multi-window algorithms. In adults with traumatic brain injury, the multi-window method improved identification of the optimal cerebral perfusion pressure (CPPopt) with the most robust autoregulation. However, multi- and single-window CPPopt had comparable associations with neurologic outcome (14). In the current study, the single-window technique outperformed the multi-window technique with wHVx. Single-window wHVx identified MAPopt values associated with injury in the paracentral gyri, basal ganglia, thalamus, and brainstem in the multivariate analysis. The MAPopt from multi-window wHVx did not show any relationships with brain injury.

### Clinical Considerations

Dysfunctional autoregulation is known to be associated with brain injury in newborns with HIE (35, 41, 42). We previously showed that having blood pressure below MAPopt is associated with greater brain injury on early MRI and poorer 2-year neurodevelopmental outcomes (4–6, 16). In those studies, we identified MAPopt using correlation HVx in 6–68-h recording durations. However, evolving brain injury (43, 44), cerebral edema (45), and hypercapnia (46) can shift the blood pressure limits of autoregulation. Averaging MAPopt across long periods of time might mask potential variation from acute clinical changes. Moreover, clinicians may need to identify MAPopt in neonates who have been monitored for only short periods. Therefore, we tested 3-h periods in the current study to advance the clinical potential of autoregulation monitoring. We also adjusted the analyses for sex, vasopressor use, seizures, PaCO_2_, and the perinatal insult score because they affect autoregulation and brain injury (15, 23, 29–33).

Our study used a time-frequency decomposition method to calculate wHVx within a coherence spectrum between MAP and rTHb. Alternative wavelet autoregulation methods use frequency-only decomposition with only one coherence spectrum. The hemoglobin volume phase index (HVP) is one example of wavelet autoregulation monitoring that uses only frequency decomposition and a specific spectral coherence identified by multivariate autoregressive models. The HVP identified an association between dysfunctional autoregulation and brain injury on MRI, developmental delay, or death (35). Additional research is needed to identify potential prognostic differences between these related but different wavelet methods.

Because our study was observational, we cannot infer whether clinically targeting MAPopt would affect the risk of brain injury. We also cannot assume a cause-and-effect relationship between autoregulation and brain injury from our data. Hemodynamic instability from severe brain injury itself may be responsible for blood pressure instability around MAPopt. This is likely reflected in the association that we identified between brainstem injury and MAPopt. Randomized clinical studies are needed to test whether optimizing autoregulation reduces brain injury in neonates with HIE.

Most neonates had moderate HIE according to the modified Sarnat staging and no or mild regional brain injury on MRI. We included five neonates who were initially diagnosed with moderate HIE at an outside hospital but were later diagnosed with mild HIE upon transfer to our NICU and during active therapeutic hypothermia. Because the clinicians decided to continue administering therapeutic hypothermia, these neonates met study criteria. Additional research is needed in a more diverse and larger population with greater HIE severity.

## Limitations

We acknowledge several limitations to our pilot study. This was a single-center study in a small cohort of neonates. Additional studies with a larger sample size are needed to study the neurologic effects of having blood pressure above or below MAPopt and to identify the best methods for finding MAPopt in neonates. The smaller sample sizes of neonates with MAPopt identified during rewarming and rewarming + normothermia and the limited number of infants with moderate to severe brain injury may have left us underpowered to detect differences. We used autoregulation indices derived from NIRS rTHb, which theoretically should be less affected by changes in cerebral metabolic rate than indices based solely on oxyhemoglobin. Nonetheless, the predominant venous contribution to the NIRS signal limits the granularity of NIRS autoregulation monitoring. We only studied blood pressure autoregulation and could not account for additional factors that influence brain injury, including metabolic derangements and inflammation. Long-term neurodevelopmental outcome data were not available for all neonates. We adjusted our analyses for several clinical factors that are known to affect autoregulation and brain injury in HIE. Though we adjusted for seizures, we did not adjust for specific anti-epileptic medications.

Finally, our findings may not be generalizable because we studied a small sample size from a university-based NICU. Only neonates with arterial catheters could undergo autoregulation monitoring. Additional selection bias could also have occurred when cases were excluded because study consent was not obtained, the baby died before autoregulation monitoring or MRI, the baby was transferred to another intensive care unit for extracorporeal membrane oxygenation, or the MRI had motion artifact.

## Conclusion

In neonates with HIE and upper MAP limits of approximately 50–60 mmHg during hypothermia, greater blood pressure above the MAPopt from single-window wHVx was associated with less injury in paracentral gyri, basal ganglia, thalamus, and brainstem. Wavelet HVx, which is a frequency-specific metric, improved the identification of neurologically relevant MAPopt values in short monitoring durations. Wavelet techniques have potential to improve neonatal autoregulation monitoring.

## Data Availability Statement

The raw data supporting the conclusions of this article will be made available by the authors, without undue reservation.

## Ethics Statement

The studies involving human participants were reviewed and approved by the Johns Hopkins University Institutional Review Board. Written informed consent to participate in this study was provided by the participants' legal guardian through May 2013. After this date, the requirement for written consent was waived.

## Author Contributions

XL analyzed the data, interpreted the data, and wrote and edited the manuscript. AT and BS analyzed the brain MRIs, interpreted the data, and edited and approved the manuscript. JP conducted the data analysis, interpreted the data, and edited and approved the manuscript. AM, RG, and KB interpreted the data, and edited and approved the manuscript. RC-V, FN, MC, and CP assisted with data collection, data interpretation, and edited and approved the manuscript. JL designed the study, collected the data, interpreted the data analysis, and wrote the manuscript. All authors contributed to the article and approved the submitted version.

## Conflict of Interest

JL, FN, and RC-V received research support from Medtronic for a separate study. JL was previously a paid consultant for Medtronic and JL is currently a paid consultant for Edwards Life Sciences. These arrangements have been reviewed and approved by the Johns Hopkins University in accordance with its conflict of interest policies. Medtronic and Edwards Life Sciences had no role in the design of the current study, collection or analysis of the data, interpretation of the results, manuscript writing, or our decision to submit this manuscript for publication. Some methods used to measure and monitor autoregulation as described in this manuscript were patented by The Johns Hopkins University, listing KB as a co-inventor. These patents are exclusively licensed to Medtronic Inc. and KB received a portion of the licensing fee. The remaining authors declare that the research was conducted in the absence of any commercial or financial relationships that could be construed as a potential conflict of interest.
