# Supplementary material for: Wavelet Autoregulation Monitoring Identifies Blood Pressures Associated With Brain Injury in Neonatal Hypoxic-Ischemic Encephalopathy
Source: Front Neurol. 2021 Apr 28;12:662839. doi: 10.3389/fneur.2021.662839 (PMC8113412; doi:10.3389/fneur.2021.662839)
Supplement: Supplementary file 1 [file Data_Sheet_1.pdf]

Supplemental Details on the Methodology.

1. The rTHb was detected at 805 nm, which is isosbestic to oxy- and deoxyhemoglobin, using 1–(optical density A) from the NIRS output to obtain a surrogate measure of cerebral blood volume (9).

2. For the wavelet hemoglobin volume index (wHVx) calculation, we used a wavelet transform coherence threshold of 0.46 to identify reliable phase shift data and reject unreliable data according to our published methods (26). Individual phase shift values with coherence higher than 0.46 were analyzed, and data points with coherence lower than 0.46 were excluded.

3. The edge effect of the morlet wavelet is $2s\sqrt{2}$, where s equals 1/f (28,29). In our study, we used the frequency range of 0.007-0.05 Hz, resulting in an edge effect of $2*\sqrt{2}$ * (1/0.05) to $2*\sqrt{2}$ * (1/0.007) seconds, or an edge effect of approximately 56 to 404 s.

Supplemental Table 1. Perinatal insult score

Parameter	2 points	1 point	0 points
**Emergency delivery [a]**		Yes	No
**Apgar score at 10 min [b]**		≤ 5	>5
**pH from the umbilical cord or arterial blood gas from 1st hour of life**	≤7.00	7.01 to ≤7.15	>7.15
**Base deficit from the umbilical cord or arterial blood gas from the 1st hour of life**	≤ -16	-15 to ≤ -10	> -10
**Sarnat stage**	3	2	1
**Mechanical ventilation**		Yes	No

[a] Emergency delivery refers to unscheduled deliveries (e.g., delivery for fetal distress).

[b] If the Apgar score was not available, the baby received 1 point if mechanical ventilation was provided.

Table reprinted with permission from Lee JK, Poretti A, et al. Optimizing cerebral autoregulation may decrease neonatal regional hypoxic-ischemic brain injury. *Dev Neurosci* 2017:39:248-256, by S. Karger AG, Basel, Switzerland.
